# The Interplay of Genetic Predisposition, Circadian Misalignment, and Metabolic Regulation in Obesity

**DOI:** 10.1007/s13679-025-00613-3

**Published:** 2025-03-01

**Authors:** Sajal Kumar Halder, Girish C. Melkani

**Affiliations:** 1 Department of Pathology, Division of Molecular and Cellular Pathology, Heersink School of Medicine, The University of Alabama at Birmingham, Birmingham, AL 35294 USA; 2 UAB Nathan Shock Center, Birmingham, AL 35294 USA

**Keywords:** Genetic obesity, GWAS, Circadian rhythm, Metabolic dysfunction, Time-restricted feeding, Physical activity

## Abstract

**Purpose of Review:**

This review explores the complex interplay between genetic predispositions to obesity, circadian rhythms, metabolic regulation, and sleep. It highlights how genetic factors underlying obesity exacerbate metabolic dysfunction through circadian misalignment and examines promising interventions to mitigate these effects.

**Recent Findings:**

Genome-wide association Studies (GWAS) have identified numerous Single Nucleotide Polymorphisms (SNPs) associated with obesity traits, attributing 40–75% heritability to body mass index (BMI). These findings illuminate critical links between genetic obesity, circadian clocks, and metabolic processes. SNPs in clock-related genes influence metabolic pathways, with disruptions in circadian rhythms—driven by poor sleep hygiene or erratic eating patterns—amplifying metabolic dysfunction. Circadian clocks, synchronized with the 24-h light–dark cycle, regulate key metabolic activities, including glucose metabolism, lipid storage, and energy utilization. Genetic mutations or external disruptions, such as irregular sleep or eating habits, can destabilize circadian rhythms, promoting weight gain and metabolic disorders.

**Summary:**

Circadian misalignment in individuals with genetic predispositions to obesity disrupts the release of key metabolic hormones, such as leptin and insulin, impairing hunger regulation and fat storage. Interventions like time-restricted feeding (TRF) and structured physical activity offer promising strategies to restore circadian harmony, improve metabolic health, and mitigate obesity-related risks.

## Introduction

Obesity, currently acknowledged as a pandemic, poses a challenge to global health, reflecting trends commonly associated with contagious diseases rather than a non-transmittable situation [1]. Like infectious diseases, obesity also affects socioeconomic groups, mostly retraining the food and health facilities. In the health sector, it always places a significant burden contributing to Noncommunicable diseases (NCDs) like cardiovascular diseases or type-2 diabetes. Obesity wreaking havoc on healthcare systems and economies, exacerbated by comorbidities like cardiovascular disease and type 2 diabetes [2, 3]. The reported 65–80% of the cases related to obesity or overweight results in type-2 diabetes. In 2022, 2.5 billion adults were overweight, with over 890 million currently residing with obesity. This represents a substantial increase from 1990 when only 25% of adults were overweight. It is estimated that the global economic consequences of overweight and obesity will exceed US$ 18 trillion by 2060 and reach US$ 3 trillion annually by 2030. The current scenario could lead to half of the world population being obese or overweight by 2035 [4]. Overall, obesity and its associated conditions directly or indirectly impact the healthcare sector.

The previous three decades have seen a more than twofold increase in the prevalence of obesity worldwide, having a substantial impact on healthcare systems, public health, and economic stability [5]. In the 1990s, research focused primarily on genetic variants influencing obesity-related syndromes, but has since evolved into extensive genome-wide studies revealing both genetic and environmental contributions to this complex condition [6]. By the mid-1990s, efforts were embedded to find genes that are associated with obesity. Much focus was on the pathways the genes were involved in and how they affect the phenotype. The term "phenotype" describes physiological, behavioral, or physical characteristics influenced by genetic makeup. Finding variants like Single Nucleotide Polymorphisms (SNPs) that influence characteristics like BMI, metabolic responses, or fat distribution is a key component of obesity research. This approach helped to find suitable gene candidates associated with different pathways leading to increased susceptibility to obesity [7]. Most recently, advanced tools have been developed to identify the SNPs of the genes through Genome-wide association Studies (GWAS) and exome sequencing [8]. This GWAS platform utilizes genotyping arrays like Applied Biosystems Axiom Array Illumina Infinium Assay and Next-Generation Sequencing (NGS) to uncover high-resolution variants of genes [9]. These innovative approaches have opened a new beginning of research where researchers are investigating the association among food, metabolism, genetic risk factors, and genetic polymorphism. Genetics is associated with a 40–75% variation in BMI population [10]. Advanced studies like GWAS and exome sequencing have substantially discovered a plethora of SNP related to BMI that could be useful for understanding the underlying cause of obesity in terms of pathophysiological condition [11]. The UK Biobank and Genetic Investigation of Anthropometric Traits (GIANT) Consortium is one of the two well-established platforms that have provided GWAS meta-analyses detecting around 941 SNPs that account for 6% BMI variance [12] which necessitates more experiments in this field. The current data from GWAS associates that genetic variants of BMI could lead to obesity-related phenotypes [11]. Interestingly, most of the GWAS investigations on different populations indicate FTO gene SNPs as a common risk factor for various obesity-related traits [13]. Variants near rs9939609 of the FTO gene are especially related to overall obesity and anthropometric traits like BMI and waist circumference [14]. The mutation in obesity could be polygenic (most common) or monogenic depending on the genetic mutation. For example, many loci near genes (*FTO, ADCY3, and BDNF*) are polygenic and related to obesity and other metabolic diseases [15]. Similarly, some of the monogenic genes include POMC, PCSK1, LEP, and LEPR [16]. Recent research conducted in 2024 on the Iranian population discovered 16 genetic variations related to BMI, of which five are significant: ADRB3 rs4994, FTO rs9939609, ADRB2 rs1042714, IL6 rs1800795, and MTHFR rs1801133 [17]. These findings elucidate the interaction between genetic predispositions and obesity, highlighting the significance of such research in identifying novel genes [18]. The discovery of new loci linked to obesity has become practiced with advanced technology, and some examples of such SNPs with genes are shown in Table 1.

**Table 1:** Recently identified obesity-linked human genes, SNPs, and their GWAS data (*P* value ≤ 10 − 8)

Human Gene	*P* value	Population	SNP (Single nucleotide polymorphism)	References
SEC16B	1.66 × 10^ − 13	Mexican mestizos	rs543874	[19]
IFNAR2	1.30 × 10^ − 12	UK Caucasian children	rs9636867	[20]
TCF4	7.48 × 10^ − 10	UK Caucasian children	rs1631486	[20]
FOXF1	5.87 × 10^ − 08	UK Caucasian children	rs7187365	[20]
PRMT6	1.12 × 10^ − 09	UK Caucasian children	rs12408810	[20]
NEGR1	3.60 × 10^ − 27	European descent	rs2815752	[19]
TCF7L2	1.11 × 10^ − 11	European descent	rs7903146	[19]
SNAPC3	4.71 × 10^ − 10	European ancestry	rs4740619	[11]
BBS1	3.50 × 10^ − 08	European	rs12805133	[21]
ETS2	1.61 × 10^ − 08	European and non-European	rs2836754	[22]
TAL1	2.18 × 10^ − 08	and non-European	rs977747	[22]
ZBTB10	3.89 × 10^ − 08	and non-European	rs16907751	[22]
MTCH2	9.00 × 10^ − 11	Japanese	rs10838738	[23]
CELF2	1.10 × 10^ − 08	European ancestry	rs10838725	[24]
ADCY3	1.79 × 10^ − 21	European descent	rs713586	[19]
GALNT10	9.99 × 10^ − 09	European and non-European	rs7715256	[22]

Apart from the genetic aspect, rising obesity rates are also a direct result of the sedentary lifestyles and high-calorie diets that have been popularized by urbanization and globalization [25]. The crisis is fueled by the interplay of genetic, behavioral, and environmental factors [26]. Behavioral factors such as diet, physical activity, and sleep patterns, along with environmental influences like toxins and obesogenic conditions, contribute to obesity alongside genetic factors. [27]. Many chronic conditions, including diabetes, hypertension, and cardiovascular diseases, not only increase the risk of obesity but have also been rising alongside the obesity epidemic. This trend places a heavy burden on healthcare systems and leads to significant financial losses. [26]. For example, around 50% percent of type-2 diabetes results from obesity in the United States [27]. Obesity also affects overweight-related cardiovascular diseases which have tripled from 1999 to 2020 in the USA [28]. Furthermore, previous studies have shown a connection between obesity, metabolic syndrome, and sleep activity. A recent clinical trial at the Mayo Clinic in Rochester, USA, involving 12 healthy, non-obese participants, found that restricted sleep combined with unrestricted food intake led to weight gain compared to the control group, which had standard sleep duration [29]. Besides the sleep period, dysregulation in the sleep–wake cycle is shown to induce obesity- causing a decrease in energy expenditure, intake of high-calorie foods, and imbalance of appetite hormones in the body [30]. All of this evidence is alarming the society to dig deeper to explore the relationship between obesity, metabolism, and sleep–wake cycle [18]. The necessity for comprehensive, long-term plans to combat obesity is increasing as countries are battling with the health and financial consequences of this epidemic [31]. Beyond traditional medication, we need to use behavioral interventions to prevent obesity. One such intervention could be time-restricted feeding/eating (TRF/TRE, known as TRE in humans) which confines food intake to a set time window, aligning with circadian rhythms to enhance metabolic health and prevent obesity. Moreover, physical activity could be another alternative therapy to improve metabolic conditions and obesity [32]. Recent studies revealed that TRE could lead to improved glycemic rate, reduced body weight, and fat reduction in controlled clinical studies [33]. Moreover, a new study from the University of Oklahoma Health Sciences Center reported that TRF/TRE also reduced white adipose tissue and pro-inflammatory markers in various adipose tissues [34]. Similarly, a randomized trial by Hunter and the group described 80 min/week of resistance or aerobic physical activity had a positive effect on preventing body weight [35]. Thus, the combined effect of TRF/TRE and physical activity could turn out to be behavior interventions for weight gain and metabolic dysregulation [36].

**Fig. 1:**
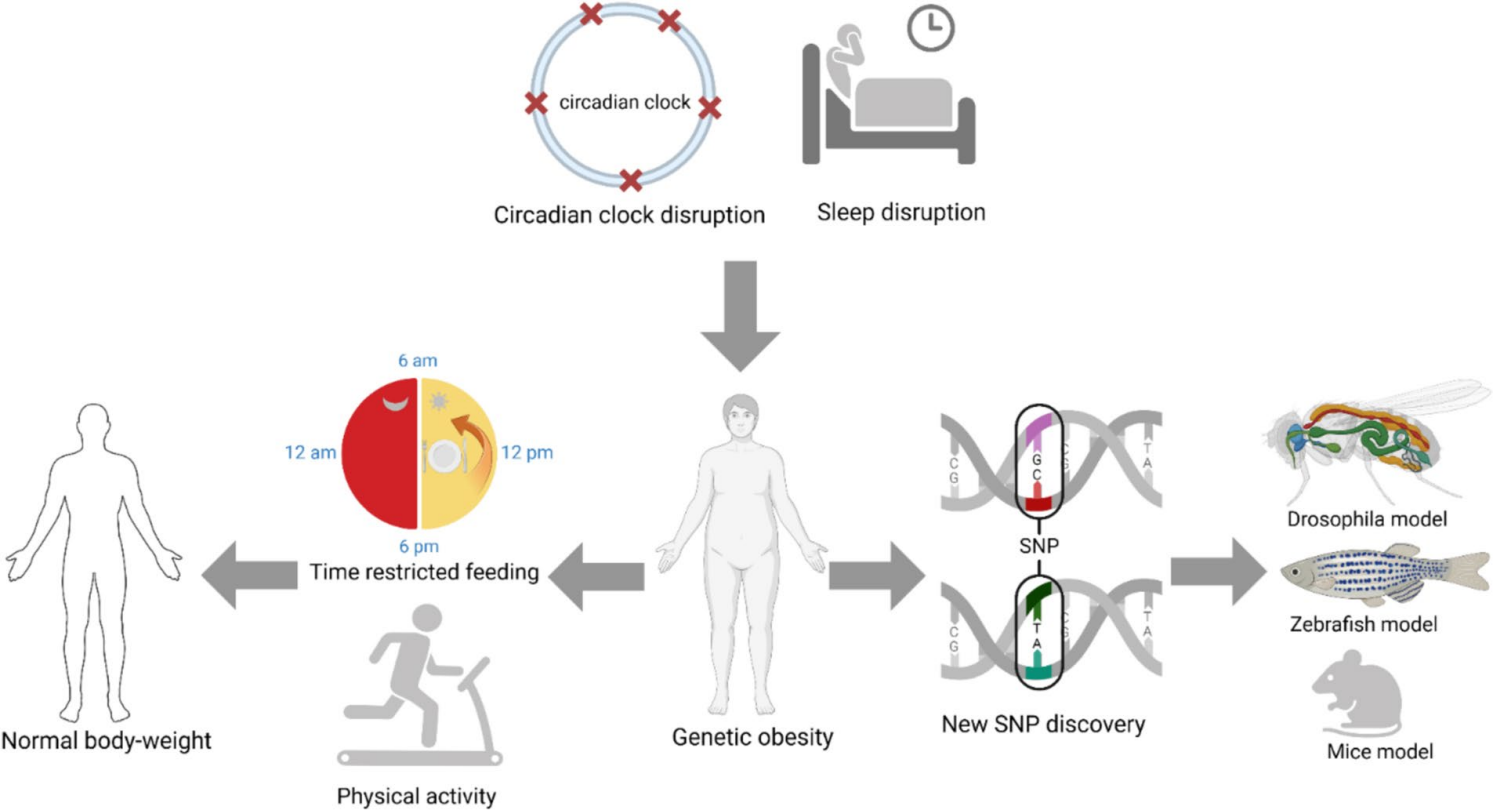
Interrelationships between circadian rhythm disruptions, sleep disturbance, and genetic factors contributing to obesity, utilizing various experimental models. At the top, the diagram shows the circadian clock marked by a circle and red crosses indicating disruption, which leads to sleep disturbances depicted by a bed icon. Left-side lifestyle interventions where normal body weight is maintained through time-restricted feeding (shown by the clock indicating mealtimes at 6 am, 12 pm, and 6 pm) and regular physical activity (symbolized by a running figure). The right side represents the genetic predisposition to obesity. A silhouette of a human figure highlights the genetic underpinnings, with emphasis on the discovery of new SNP, which are fundamental in understanding genetic obesity. (Created in BioRender. Halder, S. (2025) https://BioRender.com/f78d594)

The primary goal of this review is to examine the genetic basis of obesity and its connection to sleep-circadian rhythms and metabolic dysregulation while exploring potential therapeutic approaches. The first section discusses genetic factors contributing to obesity, followed by an analysis of how these factors influence metabolic function and sleep behaviors. Finally, we conclude with innovative strategies to mitigate obesity-related complications, aiming to reduce treatment’s financial and social burden on the broader population (Fig. 1).

## The Genetic Architecture of Obesity: Emerging Trends and Perspectives

### Elucidating Genetic Factors in Obesity

In most cases, around 40–50% of people account for the heritability of our body weight. The rate could increase up to 60–80% for obese people. There are three categories of genetic obesity: polygenic, monogenic, and syndromic [18]. Monogenic obesity is a rare form of obesity caused by a mutation in a single gene. It accounts for 5% of obesity in obese individuals [37]. This causes early-onset obesity, severe hyperphagia (a strong feeling of hunger), and in certain instances, endocrine complications [38]. The most prevalent genes linked to monogenic obesity are leptin (LEP), leptin receptor (LEPR), and melanocortin 4 receptor (MC4R) genes [39]. These genes function as a crucial regulator in controlling appetite and energy metabolism [39]. Multiple obesity-causing genetic variants coexist to cause polygenic obesity which is quite common in humans. The polygenes or polygenic loci are identified using GWAS investigations, where a p-value of ≤ 5 × 10 − 08 indicates a statistically significant SNP [1, 40]. On the other hand, syndromic obesity is a type of obesity that emerges because of a known genetic condition, which is commonly accompanied by abnormalities in organs or developmental disorders. This form of obesity is accompanied by developmental delays, intellectual disabilities, and other physical dysfunction [4]. *Cohen syndrome, Albright hereditary osteodystrophy, Bardet-Biedl, and Prader–Willi* syndromes are associated with obesity [41, 42]. There could be hundreds of these variants close to or within each gene; although individual genes may have little effect compared to monogenic ones, the significance of these variants for genetic research is immense [18]. Monogenic obesity is primarily influenced by genetics and has a limited impact on the environment. In contrast, polygenic obesity is caused by numerous genetic factors and the environment plays a significant role in determining its development and progress [18].

### GWAS Linkage to Genetic Obesity

Both genetic and environmental variables contribute to the complexity of obesity [43]. Over the last decade, there have been considerable advances in identifying genetic determinants of obesity using GWAS and exome sequencing, highlighting a complex interaction of genetic variables that contribute to this health concern [44]. In recent years, there has been an improvement in creating large sample sizes to aid in detecting abundant variants in complex genetic traits [45]. Besides, newly developed methods like Polygenic Risk Scores (PRS) help scientists detect certain risk factors from complex genetic variants which are helpful in the field of personalized medicine [46]. Advances in high throughput sequencing methods lead to identifying non-coding areas detected by GWAS [47]. Furthermore, epigenomics and transcriptomics data are being integrated into the GWAS study which also allows researchers to show the complete nature of genetic variability in complex traits [47]. This progress is fueling to identification of genetic predispositions to obesity is crucial for understanding the interplay between genetics and environmental factors, enabling personalized medicine approaches like dietary recommendations and lifestyle changes, and predicting and preventing metabolic disorders like hypertension cardiovascular diseases, and muscle dysfunction [48–51]. Recent advancements in genetic studies have led to numerous discoveries, necessitating further research. For example, researchers at the University of Exeter have shown that individuals who have a genetic variation that prevents the SMIM1 gene from functioning have larger body weights because they use less energy when they are at rest [52]. Researchers from Fudan University in China have identified a specific subgroup of mitochondrial DNA, known as M7b1a1 that is prevalent in southern China and Southeast Asia. This subgroup has a protective effect against obesity [53]. Out of these variants from M7b1a1, variant T12811C causes the change of amino acid (Y159H) in the ND5 gene that could reduce energy usage and increase heat generation. This phenomenon could lead to a decline in weight gain [54]. Extensive GWAS studies have found 941 loci that are linked to adult BMI (Body Mass Index), which is 5% of all trait variation [55]. BMI trait is polygenic and is influenced by other genetic variants and environmental factors like diet or physical activity. The low percentage indicates a low portion of variations in BMI and thus, BMI could be affected by other genetic and environmental cues [56]. The genetic background of childhood BMI is less clear, with only 15 loci linked to it, which is 2% of all the variation in 35,668 children [55]. Research indicates that obesity is polygenic, with several loci influencing the condition and each contributing a minor portion to the total genetic risk [50]. The majority of loci are located in non-coding sections of the genome, which makes it more difficult to identify the genes that are responsible and requires additional functional research to comprehend their significance [57]. For example, the FTO gene locus has been in the non-coding zones, and variants involving this region affect the level of expression of metabolic and energy-related genes residing nearby [58].

To date, researchers have identified over 1100 loci associated with obesity traits through GWAS experiments [50]. On the other hand, the finding of 25 shared loci of European and Asian cohorts is just one example of how multi-ethnic GWAS research involving populations from Europe, Asia, Africa, and Latin America has improved the genetic mapping of loci [59, 60]. However, determining the overall structure of obesity-linked variants requires representation from more non-European population-based GWAS investigations. Adding diverse biological groups could lead to identifying novel loci and improving genetic variation. Besides, multi-ethnic experiments could also narrow down those regions associated with traits and establish proper linkage disequilibrium across multiple groups with improved fine mapping [61, 62]. Similarly, a comprehensive understanding of obesity requires the integration of all genetic factors, which in turn requires a large sample size to detect rare and low-frequency variants. These variants may have a significant impact in comparison to a regular variant [63]. This could be due to less selection burden by natural selection. Some of the studies include evidence of rare genetic variants causing obesity. For example, rare variants in the DYRK1B gene were found to be responsible for monogenic obesity with type-2 diabetes [64, 65]. Like GWAS, Exome sequencing can cover rare obesity-linked variants associated with obesity [66]. Moreover, the HTR2A gene is responsible for regulating post-synaptic serotonin signaling which modulates our appetite and hormone secretion, and the SOCS3 gene is involved in negatively modulating JAK/STAT pathway by binding by attaching with the leptin or insulin [67]. Recently, one GWAS on European Americans found a relationship between BMI and several genes, notably HTR2A (rs912127) and SOCS3 (rs4969170), where HTR2A is mostly linked with males [68]. Likewise, the American population indicates that WDTC1 is associated with BMI and obesity [69]. In the Asian context, a GWAS study from a Japanese sample found two significant obesity-related variants in SEC16B (rs10913469) and GNPDA2 genes [70]. Both SEC16B and GNPDA2 genes are associated with energy storage regulation. SEC16B aids in transporting appetite hormones in the endoplasmic reticulum (ER) to the Golgi apparatus while GNPDA2 encodes hexosamine biosynthesis that is involved in metabolic regulation [23]. Similarly, on Mexican mestizos, researchers found a link between obesity and various genetic variants like NEGR1, MTCH2, and SEC16B which are also prevalent in other ethnic populations [71]. These studies showed the importance of advanced genetic studies like GWAS and how these findings are useful for future directions like finding real-world functional studies, behavioral assays, and therapeutic studies.

### Linkage of Genetic Obesity With Circadian Rhythmicity

#### Circadian Rhythm: Integrating Central and Peripheral Systems

The circadian clock framework integrates the peripheral and central neural systems, which collectively control the metabolic rhythm of the body. Situated in the suprachiasmatic nucleus (SCN) of the hypothalamus, the central clock acts as a master pacemaker by synchronizing body rhythm with the external environment and regulating the peripheral clock by SCN [72]. On the other hand, the peripheral clock resides in the liver, heart, lungs, and skin, regulating tissue-specific requirements and optimizing local circadian rhythm [73]. The periodic rotation of the earth produces 24 h of light and darkness, allowing animals to adjust to these cycles. The circadian rhythm inherent in animals and plants contributes to the regulation of physiological processes. These processes encompass metabolism, heart pumping, sleep patterns, brain function, temperature regulation, and other physiological functions [74, 75].

The system modulates food metabolism by controlling the expression of enzymes and hormones like insulin or cortisol [76, 77] which shows circadian oscillation [78]. The central clock regulates peripheral clocks via neuronal, humoral, and additional cues [79]. The regulation system controls neuronal factors including neurotransmitters or neuropeptides like gamma-aminobutyric acid (GABA), arginine vasopressin (AVP), humoral factors like cortisol, and other factors like body temperature or fasting [80, 81]. The animal circadian network comprises two primary oscillators: the central clock, which regulates synchronization to everyday light–dark periods, and the dietary-entertainable circadian oscillator, which induces activity movements through food synchronization with regularly scheduled meals [82]. Nutrient metabolism is regulated by interrelated transcriptional mechanisms that are synchronized with the circadian clock and responsive to temporal changes [83]. Food anticipatory rhythms are modulated by a food-entertainable oscillator (FEO), a particular type of the circadian clock, and modifications in clock genes are not harmful to the expression of food anticipatory mechanisms in the human body [84].

Genetic factors impact obesity by interacting with circadian rhythms that in turn regulate metabolic functions, energy balance, and dietary habits. These rhythms are also impacted by external factors like irregular light and food consumption [85]. The functioning of the circadian clock involves the synchronization of its primary clock found in the SCN, which regulates the peripheral clocks in different tissues like the liver, adipose tissue, and gastrointestinal tract through neurological and hormonal signals [86]. The role of clock genes such as Cryptochrome circadian regulator (CRY1-2) (Cryptochrome circadian regulator), Period Circadian Regulator (PER1-3), Circadian locomotor output cycles kaput (CLOCK), and Brain and muscle Arnt-like protein-1 (BMAL1) is maintaining circadian rhythms and any disturbance in those genes could induce metabolic imbalances like obesity [86]. Pancreas-specific Bmal1-null mice showed glucose tolerance that could lead to diabetes. Similarly, Bmal1-null mice represented disturbances in glucose metabolism. Both instances revealed the importance of these genes in metabolism and obesity [87, 88]. Besides, the researchers showed adipocytokines like visfatin, resistin, and adiponectin have circadian rhythmicity which are cytokines released by adipose tissue, where resistin and adiponectin represented peak and trough at Zeitgeber Time (ZT 12), lights off, and ZT 0, lights on respectively and Visfatin had a peak and trough phases at ZT 12 and ZT 24 [89]. Dysregulation in those proteins could induce obesity-related syndromes [90, 91]. Likewise, sleeping and eating habits are crucial for synchronizing the body’s peripheral clocks and maintaining the metabolic balance in the body.

### Role of Circadian Genes in Metabolic Process and Obesity

Circadian genes are essential for maintaining energy balance and regulating metabolic functions. For example, both CLOCK and BMAL1 induce the expression of several clock genes (PER and CRY) by binding to DNA as a heterodimer [92]. CLOCK genes have multiple functions like chromatin remodeling except for gene expression. Besides, genetic mutants in CLOCK genes have been reported to be associated with metabolic syndrome and obesity in CLOCK mutant mice [86, 93]. The PER-CRY gene complex act as a negative regulator for CLOCK-controlled genes that maintain the body’s circadian clock system [94]. They suppress the transcriptional ability by binding with BMAL1-CLOCK heterodimer. The combined activity helps maintain appropriate physiological activity in parallel with the 24-h day-night period [94]. One of the crucial components of this system is REV-ERBα and RORα gene regulation. While REV-ERBα inhibits the BMAL1 gene, RORα generally activates it. This oscillatory genetic activity controls the circadian clock [95]. Similarly, both PPARα and PPARγ also regulate Bmal1 and Rev-erbα core components [96]. To continue this intricate process requires a highly regulated system. Otherwise, the mutations in the circadian clock genes (CLOCK and BMAL1) could induce vulnerability to obesity. Alternatively, mutations in the circadian clock genes (CLOCK and BMAL1) could raise a predisposition to obesity [97]. Circadian genes are essential for managing obesity as they control metabolic processes and offer targets for therapy by studying the genetic and environmental factors that affect circadian rhythms. Therefore, it is imperative to conduct further investigation to uncover unexplored genes in animal models and their intricate genetic interactions, and positive or negative effects among these genes. This could lead to the discovery of a complete demonstration of human genetic obesity.

 The day-night and feeding habits cycles could be disturbed by shift work. The people who work the night shift often experience an increased level of cholesterol, LDL, and BMI [98]. Due to an imbalance between the peripheral and central body clock, this metabolic complication could occur [99]. The liver is one of the major organs responsible for regulating metabolism. By regulating the CLOCK genes Like BMAL1, CLOCK, CRY1, CLOCK, and PER2 in the peripheral tissues of this organ, glucose exhibits a significant effect on the circadian clock. Thus, any disruption in the glucose homeostasis by feeding could result in a significant imbalance in our body clock. Besides, nutrients like glucose and small organic cation compounds like polyamines also act as key regulators of the circadian rhythm through metabolic activities. These polyamines could control the body clock by modulating clock proteins like PER2 and CRY1 which inhibit the core circadian genes [100]. As the interaction of these two proteins is vital for the circadian rhythm for balancing the circadian clock process, disrupting the proteins could exhibit a reverse of the circadian clock in the body by polyamines in living cells [101, 102]. One study reported that polyamine-reduced NIH3T3 cells had longer clock gene periods. Therefore, change in any regulator could influence genetic factors and induce an imbalance in the body clock and vice-versa.

### Impact of Circadian Rhythm Disruption On Obesity

Disturbances in the sleep and wake cycle could be responsible for the susceptibility towards obesity. Similarly, genetic susceptibility to obesity could be aggravated by lacking proper sleep and an unbalanced body clock that shows the necessity of maintaining a timely sleep cycle. Recent studies are revealing novel genetic factors linked to sleep disorders and their relationship with obesity. It provides innovative ideas to intervene the obesity [103]. Changes in any key steps of metabolic processes caused by an imbalance in sleep activity and circadian clock could increase the chance of genetic predisposition for overweight [104]. Research conducted at the Mayo Clinic has shown that poor sleep quality or insufficient sleep results in increased levels of energy utilization which in turn elevates the desire for food, particularly those with high calories in the participants [104, 105]. Likewise, in a human study at the University of Washington, an increased level of a hunger-inducing hormone called ghrelin and a decreased level of a satiety hormone called leptin are observed due to sleep disruption which explains the previous event [106, 107]. Those who are genetically vulnerable to obesity could develop a system that increases the appetite for food and decreases the metabolic rate [108]. Such as the MC4RF51L in mice creates poor transport functionality MC4R/Gq/11α signaling that causes obesity and hyperphagia [109].

Several newly identified genes have been implicated in linking circadian rhythms, and metabolism with genetic obesity. For example, the FAT1 gene is associated with lipid metabolism and any disturbance in this gene contributes to metabolic disorders and obesity [110]. SOCS3 has been demonstrated to exacerbate insulin resistance, a condition that is further intensified by sleep deprivation and metabolic dysregulation [111]. TCF4 influences hepatic glucose metabolism, and circadian misalignment may exacerbate metabolic disorders [112]. PRMT6 plays a role in the regulation of circadian rhythms, and its disruption is associated with metabolic dysfunctions such as obesity and insulin resistance [113]. Variants of the KSR2 gene are correlated with excessive daytime sleepiness, which is connected to obesity due to disturbances in energy homeostasis [114]. WDTC1 is essential in lipid metabolism and the regulation of body fat, as increased expression enhances glucose utilization and decreases adiposity [115]. GNPDA2 is linked to lipid metabolism and obesity, with sleep duration influencing genetic susceptibility to obesity through the leptin pathway [116]. NEGR1 plays a crucial role in systemic metabolism, with circadian disruption contributing to obesity and glucose intolerance, particularly in the context of a high-fat diet [117]. The overexpression of MTCH2 is associated with lipid accumulation and obesity via its regulation of mitochondrial function [118]. The CELF2 gene is linked to circadian regulation and metabolic characteristics, indicating a common genetic foundation between circadian rhythm and obesity in the human body [119]. Genetic variations in sleep and circadian pathways play a crucial role in metabolic regulation, contributing to obesity. These genetic disruptions, particularly when coupled with lifestyle factors like inadequate sleep, can worsen metabolic diseases, underscoring the significance of maintaining circadian alignment to avert obesity.

### Genetic Insights Into the Dual Impact of Sleep On Obesity and Metabolic Disorders

The genetic perspective provides valuable insights into the relationship between inadequate sleep and its impact on obesity and metabolic disorders [120]. Research involving both rodents and humans has demonstrated that sleep deprivation considerably impacts energy metabolism, resulting in heightened hunger and subsequent weight gain [121, 122]. Understanding the relationship between sleep and energy conservation is essential. For example, using the rodent model it has been demonstrated that rats deprived of sleep underwent notable weight loss as a result of heightened energy expenditure, even while consuming a greater number of calories [123]. In humans, sleep deprivation results in increased energy expenditure during wakefulness. It also induces alterations in hormonal balance, particularly with the satiety hormone leptin and the appetite-stimulating hormone ghrelin [105]. Inadequate sleep demands for high energy foods for the body [124]. A study conducted on shift workers found that 81% of people who work at night prefer high-calorie foods, which is one-third of working during daylight. Despite having insignificant changes in food changes during their study, it outlines differences in the choice of food habits that could result in an imbalance in the circadian clock of the body [30].

The circadian rhythm controls the endocannabinoid system that increases the number of energy metabolism receptors in brain and other organs. University of Chicago Sleep, Health, and Metabolism Center recently studied the relationship between the circadian clock and 2-AG where they found that when sleep is shortened, researchers found an increase of 2-AG that promotes hunger and appetite in those groups devoid of enough sleep [125]. Circadian rhythm, indeed, regulates the appetite system of the body and controls appetite of the body. This shows the importance of the body clock regulating food intake and obesity in the general population. Besides, there is a hormone called ghrelin that increases the appetite for food associated with hedonic feeding. In a human study, ghrelin levels rise when sleep duration is shortened to less than eight hours, which enhances the desire for sweet foods [125]. It again indicates a direct correlation between sleep deprivation, increased appetite, and weight gain.

**Fig. 2:**
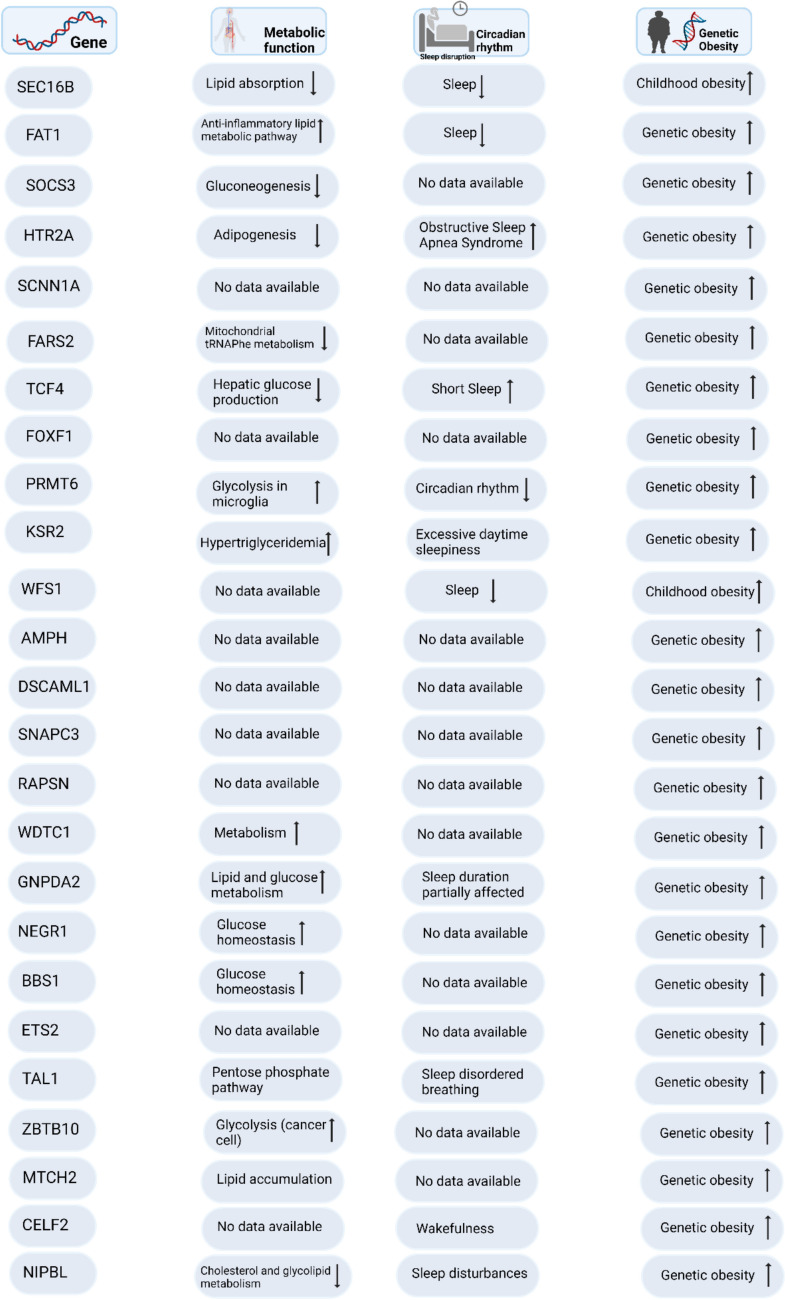
Summary of newly identified genes from GWAS investigations and their associated roles in metabolic functions, circadian rhythm disturbances, sleep patterns, and the predisposition to genetic obesity. The gene column lists genes implicated in obesity and other related metabolic disorders. The metabolic Function column describes the specific metabolic or physiological role associated with each gene, such as lipid absorption, glucose metabolism, or specific pathways like glycolysis. The sleep column indicates the impact of gene expression on sleep-related issues, such as sleep duration, wakefulness, and sleep disturbances. The genetic Obesity column shows the relationship between gene expression and genetic predisposition to obesity. Symbols like upward arrows indicate an increase in the tendency towards obesity when the gene is expressed. (Created in BioRender. Halder, S. (2025) https://BioRender.com/b76w304)

Figure 2 demonstrates the role of genetic factors identified through GWAS in influencing obesity by impacting circadian rhythms and metabolic pathways. The CLOCK and BMAL1 proteins create a heterodimer that attaches to DNA, facilitating the transcription of essential genes including PER, CRY, Rev-erb, and RORα [126]. The regulation of processes such as glucose and lipid metabolism by these genes is essential for sustaining metabolic balance. Genetic factors such as SEC16B, NIPBL, TAL1, HTR2A, TCF4, PRMT6, KSR2, GNPDA2, and FAT1 [20–22, 24, 127] could interfere with the proper functioning of this clock-controlled system. The genetic factors involved in dysregulation negatively affect glucose and lipid metabolism, resulting in disruptions to circadian rhythms. The disruption of normal energy regulation and storage processes due to these metabolic imbalances plays a significant role in the development of genetic obesity [128, 129].

Regular eating habit aligned with the proper body clock improves the metabolic process [130]. The imbalance in the body clock could lead to irregular release of hormones like insulin or cortisol that results in disrupted insulin and glucose metabolism, and it relates to altered body weight gain in the body [131].

### Genetic Factors Coordinating Both Sleep and Obesity

Several obesity-related genes overlap with unique sleep traits [132]. These factors which overlap between sleep and obesity are implicated in various genes and pathways. One of the circadian genes that regulates sleep activity and metabolic control is the CLOCK gene [133]. According to the Jackson Heart Study, specific genetic variants are linked to sleep duration and body mass index. These variants are rs2070062, rs6853192, rs6820823, rs11726609, and rs3792603. Findings from this study have important implications for our understanding of how the CLOCK gene regulates our circadian clock, which in turn affects our sleep patterns and metabolic rate [134]. T allele from CLOCK gene (rs2070062) suggested a reduced sleep time and elevated BMI for African American groups [134]. Another GWAS found that the genes FBN2, LINC01122, and RGS6, which relate to obesity, are also linked to sleep activity in children. Additionally, it indicates that in children, both the "PAX3-FOXO1 target genes" and the "Suz12 target genes" are essential pathways that modulate both sleep and obesity [135]. Also, increased expression of UCP1 and UCP2 genes, both of which are associated with obesity, indicates an obesity threat [136].

In a genetic study involving the 9-cM genome, researchers found specific zones in chromosomes 1p, 2p, 12p, and 19p that are associated with the apnea–hypopnea index (AHI). AHI refers to evaluating the severity of sleep apnea by estimating apneas (full pause in breathing) and hypopneas (partial obstruction in airways) [137]. Interestingly, 2p, 7p, and 12p chromosomal zones are also found with BMI phenotypes [138]. One study also suggested a common pathway of genes that modulate both BMI and AHI with obesity and Obstructive sleep apnea [138]. Besides, several data showed that genes characterized by obesity and other metabolic syndromes also impact sleep activity and other syndromes associated with affected sleep. For example, the FTO gene, which is linked with BMI, is correlated with distinctive sleep patterns like sleep period, snoring, and inclination to morning sleep. Similarly, the pro-opiomelanocortin (POMC) locus has been linked to obesity phenotype, and a c.231C > A change has been reported to be subjected to premature obesity [136, 139]. Elucidating the genetic link between sleep and obesity could reveal shared genetic pathways and disease-causing mechanisms.

There is a complex interplay among the circadian clock, obesity, and sleep [140]. Overall, the genetic factors play a crucial role in modulating this complex process and understanding the coordination maintained by these disease conditions could lead to the discovery of pathophysiology of them. Genetic validation of gene involved in this obesity could speed up the process of finding potential therapeutic targets.

### Integrative Models for Studying Genetic Obesity

#### Mouse Model

The use of murine models for studying genetic obesity is also widely accepted. Due to their genetic similarity with humans, the availability of diverse mutant groups of mice and the capability to conduct environment-controlled research makes them widely used in the research community [141]. The primary categories include monogenic and polygenic models, genetically modified mice, and diet-induced obesity models [142]. Monogenic models are characterized by mutations in individual genes that lead to obesity [143]. There are several genetic models available to explore obesity-related research. Examples involve: 1. ob/ob mice, which possess a mutation in the leptin gene, resulting in obesity characterized by hyperphagia and reduced energy expenditure [144, 145]. There is a mechanism called the adipose-brain axis that leptin uses to communicate with the hypothalamus by indicating the status of energy storage for long-term usage [146, 147]. This system malfunctioned in ob/ob mice when deleting leptin, misguiding the brain to perceive fasting even with excess fat accumulation. These mice also have elevated mRNA levels of neuropeptide Y (NPY) and reduced POMC which are regulators of appetite [147, 148]. Thus, these imbalances result in obesity by elevating food consumption while conserving energy.

2. db/db mice exhibit a mutation in the leptin receptor gene, leading to impaired leptin signaling and the development of early-stage obesity [143, 145, 149]. This mutation stops activation of the JAK/STAT pathway disrupting energy homeostasis. This leads to the overexpression of NPY (stimulator of hunger) and suppression of POMC (regulator of glucose metabolism), dysregulating hunger and appetite [150]. Therefore, the db/db models manifest obese traits.

3. S/S mice were utilized to investigate leptin signaling pathways beyond the conventional STAT3 pathway, contributing to overweight and hyperphagia [151, 152]. Similar to other two mutant obese models, this model demonstrates hyperphagia and obesity [142].

Polygenic models illustrate the intricate nature of human obesity, wherein numerous genes have a contributory role [153]. These models incorporate multiple genes that resemble the complex obesity traits of humans. For example:

1. New Zealand Obese (NZO) mice demonstrate obesity and type 2 diabetes, especially in males [144, 153]. They are produced by selectively breeding the fat mice from a mixed colony and then inbreeding them to adjust the trait in the mice. They demonstrate early-onset obesity, subsequently inducing dyslipidemia, hyperinsulinemia, hyperglycemia, and hypertension like adult humans [154, 155].

2. Tsumura and Suzuki Obesity and Diabetes (TSOD) mice are defined by polygenic obesity and are associated with glucose homeostasis and metabolic syndrome (MS)[156]. They are also developed to explore type 2 diabetes and obesity by selectively inbreeding the ddY obese mice models [157, 158].

Like these two mice models, Kuo Kondo-Ay (KK-Ay) and M16 mice are also utilized in biomedical science as obesity or metabolic deregulatory models. The KK-Ay models are developed from the KK strain, altering the yellow obese (Ay) gene, whereas the M16 mice are derived from inbreeding the ICR outbred mice. While KK-Ay mice develop hyperinsulinemia, and hyperglycemia at the 8 weeks of age, the M16 mice induce early onset obesity by faster weight gaining [158, 159]. They both are useful mice models to explore the genetics of obesity models, their relationship with metabolic disorders, and test the anti-diabetic or anti-obesity drugs.

Genetically engineered models encompass transgenic and knockout mice that have been changed to investigate particular genes associated with obesity[160].

1. Transgenic animals, such as MCH-OE mice, exhibit overexpression of melanin-concentrating hormone, resulting in insulin resistance and obesity [161, 162].

2. Knockout animals like β3-AR knockout mice exhibit mild overweight attributable to diminished sympathetic nervous system function [163].

Finally, diet-induced obesity models replicate human obesity by administering high-fat or high-carbohydrate meals to mice. Animals subjected to a high-fat diet (HFD) exhibit overweight and resistance to insulin, analogous to human eating habits [144, 153].

#### Zebrafish Model

The zebrafish model utilized for investigating genetic obesity emphasizes the exploration of physiological and genetic determinants that contribute to the condition. Zebrafish are increasingly recognized as important models in obesity research, owing to their shared genetic makeup with humans, especially regarding metabolic pathways [127]. Transgenic zebrafish models are developed through the overexpression of obesogenic genes, including Agouti-related peptide (AgRP), resulting in heightened body weight, fat accumulation, and elevated triglyceride levels [127, 164]. A different model includes the alteration of microRNA miR-27b, which controls the metabolism of lipids, leading to hyperlipidemia and hepatic steatosis [165, 166]. Genetic manipulation in zebrafish is easy and effective in studying obesity and lipid metabolism. For example, Tg (hPPARγ-eGFP), Tg (lyz:Ds Red), and Tg (pck1:Luc2) lines are one of the models to explore lipid accumulation, macrophage infiltration, and gluconeogenesis respectively in adipose tissue [167]. Furthermore, zebrafish mutants such as Tg (krt4Hsa.myrAkt1)cy^18^, created for cancer research, display obesity-related characteristics including adipocyte hyperplasia, irregular fat distribution, and glucose intolerance [168]. The knockdown of the transient genes in zebrafish by Morpholinos (gene silencer) is done by researchers to study obesity [169]. For example, the knockdown of ApoC2 is accomplished to explore the lipid metabolism in zebrafish [170]. ApoC2 is responsible for activating lipoprotein lipase which is essential for breaking down triglycerides and energy storage [141]. Besides, overexpression of specific genes like AKT1 in adipocytes leads to the development of severe obesity causing glucose intolerance [171]. Serine/threonine protein kinase, AKT1 is involved in diverse pathways that regulate cell cycle, cell growth, cell differentiation, cell metabolism, and angiogenesis [172]. These transgenic models are useful for studying the complexity of genetic obesity, avoiding human involvement.

The genetic mutants of zebrafish are developed by genetic techniques like ENU mutagenesis and Clustered regularly interspaced palindromic repeats/ CRISPR-associated protein 9 (CRISPR/Cas9) [173, 174]. The genetic models illustrate the preserved control of the metabolism of lipids across zebrafish and mammals [171, 173, 174]. Mutant zebrafish lines have been essential for investigating lipid metabolism since they display phenotypes such as fatty liver and overweight resulting from mutations in genes associated with ER stress and vesicular movement [127]. For instance, the Foie gras and cdipt mutants demonstrate hepatic steatosis resulting from mutations that impact ER to Golgi transportation [175, 176], whereas others such as Red Moon (rmn) emphasize the significance of ketone body export in maintaining energy equilibrium [177]. Mutants such as vizzini (growth hormone mutation) illustrate the significance of hormonal control in fat distribution and obesity [167]. The gh1 mutation in vizzini results in reduced growth hormone and increased subcutaneous adipose tissue (SAT) droplet that indicates higher adipose tissue deposition [178]. These models assist investigators in comprehending the genetic functions underlying obesity and act as potential systems for evaluating therapeutic interventions [167].

These genetic models are useful for potential therapeutic studies in laboratory settings. Obtaining an initial insight from these models could open a gateway for conducting advanced research like clinical trials for researchers. Therefore, it is useful to develop novel model organisms to advance ongoing research. In that case, more and more genetic studies are required to explore the genetic makeup of human obesity that could be utilized in different models.

#### Drosophila Model

*Drosophila melanogaster* is a model organism excellent for studying genetic screening and elucidating the body clock [179]. They could be also useful in studying lifespan and sleep regulation due to the short life of drosophila [179, 180]. The genetic bipartite GAL4-UAS tool is one of the gene manipulation systems that help overexpress or knock down transgenic genes. This tool enables researchers to conduct functional studies by manipulating genes, time-specific gene expressions like temperature-sensitive GAL80ts, and tissue-specific control genetic manipulation [180–182]. Besides, CRISPR and TALENs (transcription activator-like effector nucleases) are two of the most popular genomes editing mechanisms widely used for studying mutant models [183, 184]. CRISPR editing allows researchers to create targeted deletion, insertion, or mutations in the genome, even in the germline to pass subsequent generations of flies, and specific base changes by temperature-tolerant CRISPR in the genome to study gain or loss of function of fly genes [185–187]. Similarly, TALENs can also be used for genetic modification in flies to insert double-strand breaks at a specific genomic location [187]. Genetic Methodologies in Obesity Research utilizing Drosophila:

RNAi Screens: In the *Drosophila melanogaster* model, RNA interference (RNAi) lines are used to categorically silence the genes of interest to explore their functions. This extensive process incorporates a double-stranded RNA (dsRNA) drosophila body, inducing the RNA interference (RNAi) cascades of the pathway leading to the decline of target mRNA. The Dicer-2 enzyme in drosophila processes the double-stranded RNA (dsRNA) into smaller interfering RNAs (siRNAs). These siRNAs are incorporated into an RNA-induced silencing complex (RISC) and used to guide the complementary mRNA sequences. This leads to the silencing of the genes by cleaving off or degrading mRNA. Thus, it effectively reduces the expression of genes [188]. Likewise, scientists have employed RNAi screens to methodically silence genes that are associated with fat metabolism and obesity. Using tissue-specific RNAi to screen many human (BMI) GWAS loci showed that 42% of the testable human BMI-associated loci showed significant fat traits when their fly orthologs were knocked down [189].

Transgenic Models: Transgenic models are instrumental in studying the genetic mechanism of obesity by manipulating the genes in Drosophila. These animal models usually bear foreign genetic materials, transgene in their genome. Scientists could study phenotypic changes in drosophila by modifying existing genes or introducing foreign genes [190]. This genetic modification could provide insight into energy homeostasis and metabolic regulation to study different mutants related to obesity. Recently, scientists have developed a transgenic obese model by expressing the human synphilin-1 in Drosophila resulting in obesity-like traits, characterized by elevated food consumption and increased body mass [191]. BAC-based transgenic reporters have been employed to investigate the expression of genes implicated in metabolic control, including the CCHamide-2 receptor (CCHa2-R) in insulin-producing cells (IPCs) within the brain [192]. The CCHamide-2 binds to CCHa2-R (receptor) and induces the production of Drosophila insulin-like peptides (Dilps) that control glucose metabolism in the fly body [192]. These could provide useful study models for developing novel therapeutics and exploring extensively on pathogenesis of obesity [191].

Mutant Strains: Genetic mutations in particular genes have been utilized to develop obesity models in Drosophila. mutations in the Split ends (*Spen*) gene lead to obesogenic phenotypes, characterized by diminished lipid catabolism and heightened glycolytic flux [193]. Besides, the mutations in Brummer (*bmm*) and Adipose (*Adp*) genes have shown increased fat storage because of defects in lipolysis and elevated fat accumulation [194].

Researchers recently developed the OBL (Obese-Like) model in the Drosophila model to study obesity-related metabolic disorders by lowering ecdysone concentrations, delaying the larval period. They mimicked the mammalian obesity traits by genetic manipulation hyperphagia, elevated lipid deposition, adipocyte hypertrophy, and increased circulating glucose [195]. This study also claimed that reducing Eiger/TNFα expression could be used for obesity, type 2 diabetes, and other metabolic syndrome models [195]. Our lab previously identified several genetic Drosophila models for pathological obesity. For example, complete or partial loss of function in the *Bmm* gene served as suitable models for obesity traits [196]. This gene is essential for lipid storage that encodes for lipid storage droplet-associated TAG lipase Brummer [197]. These new findings add adding novel model system for obesity that could be used further for therapeutic study.

All these models are essential for studying genetic obesity and its link with circadian rhythm and metabolism. Every day, newer genes are coming into the main stage which establishes the relationship strongly. Interestingly, these genes could be explored extensively to be introduced as biological models for genetic obesity. Despite progress in understanding the genetic determinants of obesity and their interactions with circadian rhythms, sleep, and metabolic pathways, these findings remain incomplete. Current models, such as those integrating GWAS findings with functional studies in animal systems, provide a valuable foundation but underscore the need for more comprehensive genetic interaction mapping. Achieving this requires the integration of multi-omics data, research across diverse populations, and advanced modeling techniques to identify rare and low-frequency genetic variants.

## Potential Future Research and Clinical Applications

### The Effects of TRF/TRE On Obesity

TRF is a growing dietary strategy to address food restricting it to 6 to 10 h a day without changing or limiting the nutritional quota in the food [198]. The implementation of the TRF regimen is known to improve glucose tolerance, metabolic flexibility, and reduction of body weight [198]. The mechanistic basis of obesity-linked has been shown using genetic models, in conjunction with omics data [196, 199–201]. For example, our group has generated diet and genetically linked obesity *Drosophila* models and shown the role of TRF in improving muscle function linked with obesity and circadian rhythm disruption [196], Furthermore, genetic validation and omics approaches reveal that TRF enhances muscle function via purine cycle and AMPK signaling pathways in Drosophila obesity models [200]. This approach has shown promising results that could be resistant to genetic inclination to obesity by modulating different cellular and metabolic pathways including the role of circadian clocks [196, 199–201]. TRF utilizes unique mechanisms to regulate these processes.

TRF could help optimize metabolic steps by synchronizing food habits with the body’s circadian clock. For example, in rodent models, TRF could upregulate the expression of primary clock genes like BMAL1, CRY1, Rev-erbα, and PER2 when they are fed high-fat food. Consequently, these genes restore glucose uptake and insulin resistance by synchronizing metabolism and energy distribution in the body’s circadian rhythm [198]. Besides, it could also decrease the excessive synthesis of glucose by stimulating gluconeogenesis. It generally elevates the level of pCREB phosphorylation (transcription factor) during starvation by reinstating the circadian cycle of the body [202].

TRF acts as a key regulator of lipid metabolism by stimulating AMP kinase (AMPK) and repressing acetyl-CoA carboxylase (ACC), which are vital modulators of fatty acid production. As the AMPK level rises, it phosphorylates ACC, making it inactive and decreasing lipogenesis [202]. Furthermore, TRF boosts the function of Rev erbα, a component of the body’s clock that inhibits genes like Fasn which is a multi-enzyme protein complex responsible for fatty acid production [203]. This coordinated regulation decreases the generation of fatty acids and promotes the breakdown of fatty acids resulting in a decrease in accumulation and inflammation in the liver [202, 203].

In the obesity model of mice, TRF has been shown to improve adipose tissue inflammation. One such study confirmed that TRF mice had little amount of adipocyte tissue with reduced infiltration of macrophage. This indicates a lower level of pro-inflammatory cytokines like TNF-α and IL-6 in the body. Consequently, it subsequently improves metabolism through this anti-inflammatory activity [204, 205]. In context with the human trial, a recent randomized 6-month clinical research having participants aged 18 to 80 years with obesity and T2D showed that a TRE strategy, devoid of calorie tracking, was efficacious for weight reduction and decreasing HbA1c levels in persons with type 2 diabetes [206]. Research on 139 patients with obesity comparing calorie restriction with and without TRE revealed that both procedures led to weight loss and a reduction in fat mass, with no significant difference observed between the two approaches [207].

Overall, TRF could reprogram metabolic pathways and the circadian clock by TRF and lead to the restoration of fat deposition in the obese body. Researchers have found early time-restricted feeding (eTRF) could increase insulin resistance, improve glucose metabolism, and decrease eating habits in the evening inducing weight loss [208, 209]. So, this could be a potential therapy to mitigate obesity.

### Physical Activity: Mitigating or Reversing Genetic Predispositions to Obesity

An important regulator that may significantly impact the risk of obesity development, even in cases of hereditary obesity, is physical activity (PA). Various studies have shown that incorporating regular physical activity into one’s daily routine may effectively control BMI and other health issues associated with obesity [210].

An analysis of 19,308 adult twins from the Chinese National Twin Registry supported the notion that physical activity can influence building up fat in the body. With taxing physical activity, the study reveals it could mitigate the genetic repercussions on the BMI of Chinese citizens [211]. A similar study engaging 47,691 participants of runners revealed the impact of the ‘total running path’ on the genetic likelihood of obesity. According to this study, the amount of running contributes to the BMI and WC, and an average running ≥ 9 km/day decreases the BMI of females by 58% and males by 48% [212]. This concludes the significance of running as a physical activity to improve metabolism and body fat. A recent study conducted on a group of 20,000 individuals as part of the EPIC-Norfolk project has found that physical activity can reduce the risk of genetically induced obesity, which is a major contributor to common obesity, by about 40%. Additionally, this study showed that PA reduced the FTO rs1121980 genotype on BMI and WC [213]. Another study on Han Chinese individuals showed that physical activity like jogging, climbing, and walking decreases the danger of genetic predisposition to obesity [214].

Therefore, both TRF and physical activity are crucial when we consider their effect on genetic traits. These studies underscore the significance of TRF and PA which are not only improving metabolic health but also enhancing the activity of muscle and other correlated diseases. So, these strategies should be popularized to tackle the obesity pandemic, particularly the inclination to genetic complications. TRF generally synchronizes food consumption with the sleep and wake cycle of the body, thereby improving health [215]. It is always pivotal for controlling the epigenetic landscape of the body through DNA methylation and histone modification, which regulate gene expression [216]. Clock genes, mainly CLOCK: BMAL1 complex coordinate epigenetic modulators by dynamic acetylation and deacetylation processes [217]. A recent study reported rhythmicity or differential expression of 80% of all the genes in at least a single tissue under TRF [218]. Through epigenetic regulation of pancreatic β cell function, TRF prevents the harmful metabolic effects caused by circadian disruption. This confirms that TRF could improve glucose homeostasis by regulating gene expression in those tissues [219]. Like TRF, physical activity can also alter gene expression by epigenetic modification. For example, endurance training is associated with DNA methylation on multiple sites of the genome, especially the enhancer sites [220] and exercise-induced histone modification could improve skeletal muscle and metabolic health [221]. There was evidence of promoter region hypomethylation of genes that can improve metabolic processes, such as fatty acid transporter and nuclear receptor factor [222]. Additionally, skeletal muscle biopsies from exercisers showed reduced methylation in the regulatory region of the PGC-1α gene, a crucial regulator of energy expenditure and mitochondrial biogenesis [223]. As a result, TRF and physical activity could be influential to significantly influence the regulation of our body clock and metabolic pathways through epigenetic modification, thereby enhancing the obesity condition.

### Limitations, Challenges, Significance, and Future Directions

Limitations of this study include addressing the complexity of genetic and environmental interactions in obesity. Integration of genetic models, including GWAS and functional animal models, is unable to provide detailed insights into the interplay of genetic variants and metabolic pathways. Also, capturing rare and low-frequency variants might have limited significance on obesity susceptibility. Furthermore, the impact of diverse environmental factors including diet, physical activity, and sleep patterns on obesity genetics is still not well understood. Additionally, existing animal models, including mice, zebrafish, and Drosophila offer promise, however, do not fully replicate the human metabolic and circadian regulatory systems, thereby not applicable directly to clinical settings. Finally, most of the genetic studies can obtain contributions of social, circadian, and behavioral determinants, while these factors directly influence obesity. While challenging, it is important to address these limitations which will require more detailed genomic methods from large but diverse populations including their integration with omics and advanced modeling techniques. Overall, these comprehensive approaches will be helpful to establish a more holistic understanding of obesity risk factors.

**Fig. 3:**
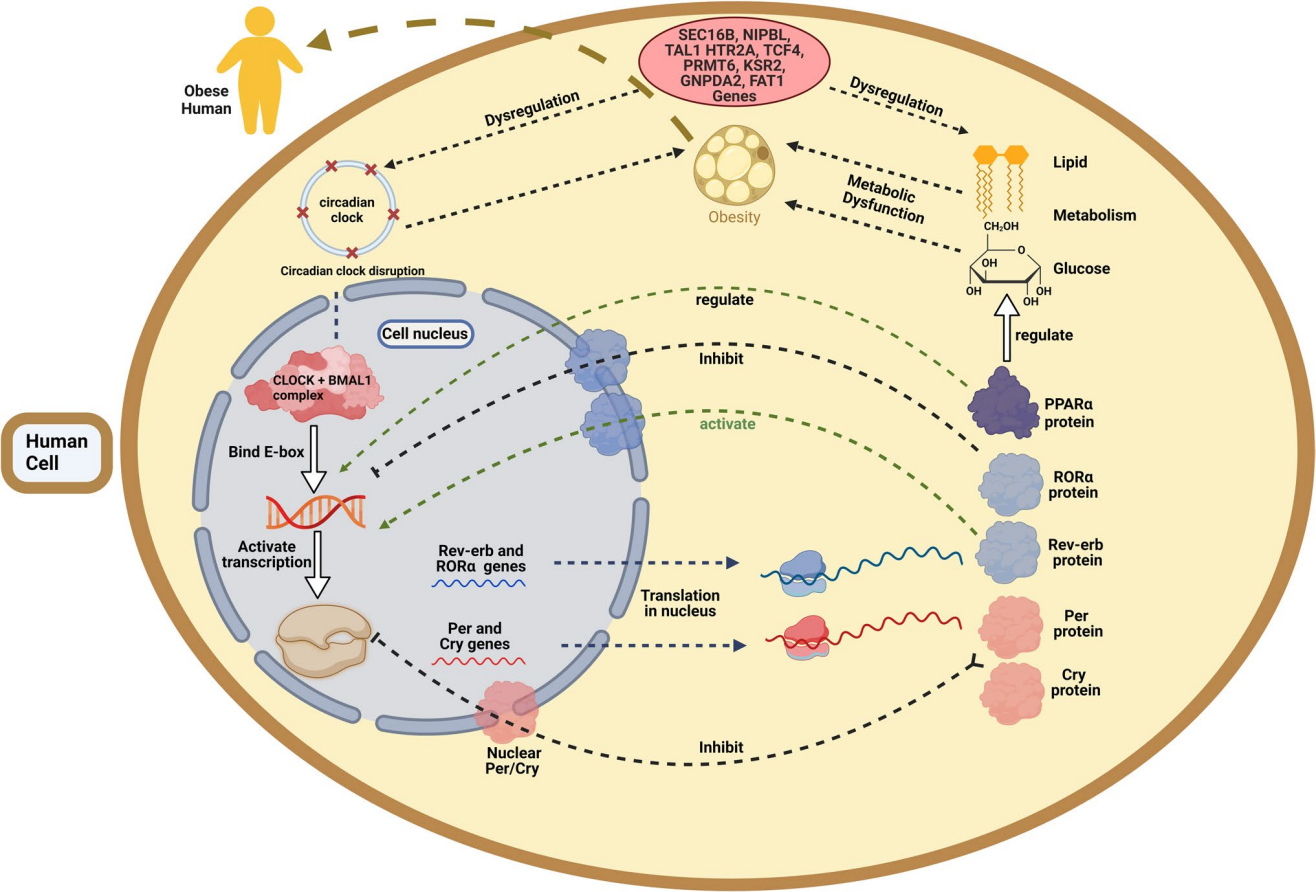
The influence of CLOCK and genetic determinants on circadian regulation and obesity. This diagram depicts the biological pathways connecting circadian clock genes, transcription factors, and metabolic regulation to obesity. The CLOCK heterodimer initiates circadian transcription by binding to E-box sites, resulting in the transcription of PER/CRY and Rev-erb/Ror genes. Per and Cry proteins exert negative feedback, whereas RORα enhances and REV-ERBα suppresses Bmal1 transcription, affecting glucose and lipid metabolism on the right. Genetic variables associated with obesity revealed by GWAS, including SEC16B, NIPBL, TAL1 HTR2A, TCF4, PRMT6, KSR2, GNPDA2, and FAT1, contribute to circadian dysregulation and metabolic dysfunction, resulting in obesity. The graphic highlights the interrelated functions of circadian rhythm disruption and genetic susceptibility in fostering metabolic abnormalities and obesity. (Created in BioRender. Halder, S. (2025) https://BioRender.com/p77a185)

In the human body, genetic obesity poses a complex condition where multiple factors like genetic variants, circadian rhythms, and metabolic processes are worsening the situation worldwide (Fig. 3). The disruption of quality sleep or metabolic homeostasis could lead to exacerbation of obesity. This review highlights the interplay between genetic variants, metabolic pathways, and circadian rhythms, underscoring their combined influence on obesity-related health outcomes. Circadian misalignment due to disrupted sleep patterns and erratic food intake exacerbates genetic predispositions to obesity. Strategies like TRF and planned physical activity offer promising interventions by synchronizing metabolic processes with the body’s natural rhythms, thus mitigating genetic risks.

Future research should focus on integrating multi-omics approaches—including genomics, epigenomics, transcriptomics, and metabolomics—to uncover novel genetic and environmental interactions. Animal models such as CRISPR-engineered mice, zebrafish, and Drosophila with circadian and metabolic perturbations could help refine our understanding of obesity-linked pathways. moreover, researchers GWAS could identify SNPs near those genes that are associated with metabolic pathways like insulin resistance, glucose, and lipid metabolism. Elucidating the relationship between these genetic factors with circadian rhythms could provide insights into the complexity of genetic obesity if integrated with efficient genetic models could provide research tools to evaluate complex interactions [196–205]. Interventions like TRF and physical activity could help synchronize eating habits with the body’s natural circadian rhythms and counteract genetic effects on BMI, ultimately improving metabolic health. Moreover, clinical studies should explore how personalized interventions based on genetic risk scores can improve metabolic health outcomes. Advances in artificial intelligence and machine learning can facilitate the development of predictive models for obesity risk assessment and personalized treatment strategies. Further, public health policies should prioritize awareness campaigns that promote TRF, physical activity, and sleep hygiene as preventive measures against obesity. Addressing these aspects will contribute to more effective, evidence-based interventions aimed at reducing the global obesity burden.

## Key References

- Xu H, Gupta S, Dinsmore I, Kollu A, Cawley AM, Anwar MY, Chen H-H, Petty LE, Seshadri S, Graff M. Integrating Genetic and Transcriptomic Data to Identify Genes Underlying Obesity Risk Loci. medRxiv 2024:2024.06. 11.24308730.
  - The GWAS data suggests that genetic variants associated with BMI may contribute to obesity-related phenotypes.
- Damavandi N, Soleymaniniya A, Bahrami Zadegan S, Samiee Aref MH, Zeinali S. Development of a genetic risk score for obesity predisposition evaluation. Mol Genet Genomics 2022;297(6):1495-503. 10.1007/s00438-022-01923-0.
  - Population-based studies identified 16 genetic variations associated with BMI, highlighting five significant ones: ADRB3 rs4994, FTO rs9939609, ADRB2 rs1042714, IL6 rs1800795, and MTHFR rs1801133.
- Covassin N, Singh P, McCrady-Spitzer SK, Louis EKS, Calvin AD, Levine JA, Somers VK. Effects of Experimental Sleep Restriction on Energy Intake, Energy Expenditure, and Visceral Obesity. Journal of the American College of Cardiology 2022;79(13):1254-65. 10.1016/j.jacc.2022.01.038.
  - A recent clinical trial at the Mayo Clinic in Rochester, USA, found that restricted sleep with ad libitum food intake led to weight gain in 12 healthy, non-obese participants compared to the control group with standard sleep.
- Broussard JL, Van Cauter E. Disturbances of sleep and circadian rhythms: novel risk factors for obesity. Curr Opin Endocrinol Diabetes Obes 2016;23(5):353-9. 10.1097/med.0000000000000276.
  -
    - Disrupted sleep-wake cycles contribute to obesity by reducing energy expenditure, increasing high-calorie food intake, and disrupting appetite hormones.
- Xie Z, Sun Y, Ye Y, Hu D, Zhang H, He Z, Zhao H, Yang H, Mao Y. Randomized controlled trial for time-restricted eating in healthy volunteers without obesity. Nat Commun 2022;13(1):1003. 10.1038/s41467-022-28662-5.
  - Time-restricted feeding and physical activity offer potential strategies to prevent obesity and improve metabolic health.
- Bao R, Sun Y, Jiang Y, Ye L, Hong J, Wang W. Effects of Time-Restricted Feeding on Energy Balance: A Cross-Over Trial in Healthy Subjects. Front Endocrinol (Lausanne) 2022;13:870054. 10.3389/fendo.2022.870054.
  -
    - This recent study showed that TRF improves glycemic rates, reduces body weight, and decreases fat in clinical trials.
- Livelo C, Guo Y, Abou Daya F, Rajasekaran V, Varshney S, Le HD, Barnes S, Panda S, Melkani GC. Time-restricted feeding promotes muscle function through purine cycle and AMPK signaling in Drosophila obesity models. Nat Commun 2023;14(1):949. 10.1038/s41467-023-36474-4.
  - The integration of Drosophila models with omics data revealed the role of time-restricted feeding in identifying shared and distinct pathways associated with genetic and diet-linked obesity.

## Data Availability

No datasets were generated or analysed during the current study.
